# Environmental exposure and the role of AhR in the tumor microenvironment of breast cancer

**DOI:** 10.3389/fphar.2022.1095289

**Published:** 2022-12-15

**Authors:** Colleen Sweeney, Gwendal Lazennec, Christoph F. A. Vogel

**Affiliations:** ^1^ Department of Biochemistry and Molecular Medicine, School of Medicine, University of California Davis, Sacramento, CA, United States; ^2^ Centre National de la Recherche Scientifique, SYS2DIAG-ALCEN, Cap Delta, Montpellier, France; ^3^ Center for Health and the Environment, University of California Davis, Davis, CA, United States; ^4^ Department of Environmental Toxicology, University of California Davis, Davis, CA, United States

**Keywords:** AhR, breast cancer, air pollution, particulate matter, environmental injustice, macrophages, tumor microenvironment, tumor promotion

## Abstract

Activation of the aryl hydrocarbon receptor (AhR) through environmental exposure to chemicals including polycyclic aromatic hydrocarbons (PAHs) and polychlorinated dibenzo-p-dioxins (PCDDs) can lead to severe adverse health effects and increase the risk of breast cancer. This review considers several mechanisms which link the tumor promoting effects of environmental pollutants with the AhR signaling pathway, contributing to the development and progression of breast cancer. We explore AhR’s function in shaping the tumor microenvironment, modifying immune tolerance, and regulating cancer stemness, driving breast cancer chemoresistance and metastasis. The complexity of AhR, with evidence for both oncogenic and tumor suppressor roles is discussed. We propose that AhR functions as a “molecular bridge”, linking disproportionate toxin exposure and policies which underlie environmental injustice with tumor cell behaviors which drive poor patient outcomes.

## Introduction—*Environmental exposure and breast cancer*

Air pollution and occupational exposure studies have reported positive associations with the risk of developing breast cancer (Amadou et al., 2021). Air pollution and ambient particulate matter (PM) contain a complex mixture of compounds, including polycyclic aromatic hydrocarbons (PAHs) and various metals (e.g. iron, nickel, copper), which may induce reactive oxygen species (ROS) and inflammation (Rückerl et al., 2007; Grunig et al., 2014) and stimulate the progression of breast cancer (Romaniuk et al., 2017). PAHs are generated during combustion processes and derive from various sources such as indoor fireplaces, wildfires, industrial activities, and vehicular traffic and the exposure to PAHs has been identified as a risk factor for breast cancer (Lichtiger et al., 2021; Gamboa-Loira et al., 2022). Importantly, a stronger association of breast cancer risk was found with traffic related air pollution (TRAP) and higher PAH exposure intensity and duration of exposure (Brody et al., 2007; Nie et al., 2007; Mordukhovich et al., 2016; Large and Wei 2017; Shen et al., 2017; Lee et al., 2019). Vehicular traffic is a major ambient source of PAH exposure and the PAH benzo[a]pyrene (BaP) is classified as a human carcinogen by the International Agency for Research on Cancer (IARC, 2010). Furthermore, BaP and other PAHs have been identified as ligands of the aryl hydrocarbon receptor (AhR), a ligand-activated transcription factor, belonging to the bHLH-PAS family, which regulates multiple target genes and is best known for its role as a xenobiotic receptor (Boonen et al., 2020; Vogel et al., 2020). The activation of the AhR signaling pathway *via* environmental pollutants including dioxins and PAHs has been associated with the development of breast cancer (Birnbaum and Fenton 2003; La Merrill et al., 2010; Murray et al., 2014; Warner et al., 2011; Kolluri et al., 2017; Donovan et al., 2018; Narasimhan et al., 2018; Gearhart-Serna et al., 2020). Reports from our team and other groups suggest an important role of AhR as an immune-modulator and mediator of toxic responses triggered by particulate matter (PM) derived from TRAP (O'Driscoll et al., 2019; O'Driscoll and Mezrich, 2018; Castaneda et al., 2018; Yuan et al., 2020).

Recent studies confirmed an increased risk of breast cancer with vehicular-specific PM exposure among African American and Japanese American women living near major roads, highlighting the link between environmental injustice and health disparities (Cheng et al., 2020; Niehoof et al., 2020). Indeed, residential proximity to major roadways is a recognized risk factor beyond breast cancer, in cardiovascular disease (Hart et al., 2014; Kirwa et al., 2014; Kingsley et al., 2015; Kubil et al., 2018) and renal disease (Lue et al., 2013). Further, it disproportionately impacts racial and ethnic minoritized groups and those of lower socioeconomic status, the legacy of the widespread practice of redlining in the United States (Hwa Jung et al., 2022; Swope et al., 2022). While the Fair Housing Act of 1968 prohibited racial discrimination in housing and lending, exclusionary zoning and other practices such as gentrification has perpetuated residential segregation (https://www.brookings.edu/research/neighborhood-segregation-persists-for-black-latino-or-hispanic-and-asian-americans/). In a study of Hillsborough County in Florida, Stuart et al. (2009) found that blacks, Hispanics, and people living below the poverty line are much more likely to reside close to sources of air pollution but further from air quality monitoring sites while whites were found to live closer to monitoring sites but significantly further from pollution sources. Wu et al. (2014) found that particulate matter (PM) collected near a major Los Angeles freeway (compared to an urban background location) induced significantly higher production of the cytokines IL-6, IL-8, and TNF-α, suggesting a link between AhR activation, AhR-driven inflammation (Vogel et al., 2011; Vacher et al., 2018; Wu et al., 2021) and proximity to pollution. The interaction between environmental exposure, socio-economic related stress and psychosocial stress in under-resourced neighborhoods has been termed the environmental “riskscape” by Morello-Frosch & Shenassa, 2006 (Morello-Frosch & Lopez, 2006). As noted by Morello-Frosch, the Institute of Medicine recognizes this as a type of “double jeopardy” in which elevated stress impairs the ability of individuals living in under-resourced neighborhoods to endure the myriad health consequences of chronic environmental exposures (https://www.scientificamerican.com/article/end-double-jeopardy/#).

## Role of AhR in breast cancer

Approximately 2 decades ago, AhR was found to be overexpressed in mammary cancer in rats (Trombino et al., 2000) sparking curiosity as to its role in breast cancer progression. Several studies have since shown that chemical exposure and AhR activation affect processes of mammary gland differentiation, disrupting pregnancy-related differentiation and milk production, and increasing the risk of breast cancer (Warner et al., 2002; Vorderstrasse et al., 2004; Lew et al., 2011; Belton et al., 2018; Kay et al., 2022). Further studies have elucidated AhR’s molecular contribution to carcinogenic progression and ratified the oncogenic role of AhR in breast cancer cells (Wang et al., 2017; Wang et al., 2020). In support of its role as a breast cancer oncogene, AhR activation is sufficient to transform human mammary epithelial cells and promote their migration, invasion and epithelial-to-mesenchymal transition (EMT) (Brooks and Eltom 2011). Work from our group showed that chronic exposure of MCF10AT1 and MCF-7 cells to estradiol (E2) resulted in AhR overexpression and downregulation of estrogen receptor alpha (ERα) and progesterone receptor (Zou and Matsumura 2003; Wong et al., 2009) accompanied by increased proliferation, invasion, and apoptosis resistance. The resistance to apoptosis was also demonstrated in human breast cancer cell lines treated with the prototypical AhR ligand TCDD when apoptosis was induced by chemotherapeutics (doxorubicin, lapatinib and paclitaxel) (Bekki et al., 2015). Treatment with PAH mixtures which bind to and activate AhR also increased cell proliferation and expression of antiapoptotic proteins in MCF-7 cells *via* AhR signaling (Gearhart-Serna et al., 2020).

Several studies have reported AhR overexpression in human breast cancer (Li et al., 2014; D’Amato et al., 2015; Vacher et al., 2018). Using samples from breast cancer patients, we found that AhR is frequently over-expressed in ER-negative human breast tumors, and this is closely correlated with elevated expression of the NF-кB subunit RelB and inflammatory markers such as IL-8 (CXCL1 in mouse) and COX-2 (Vogel et al., 2011). This was also observed by Vacher et al. with significant overexpression of cytokines, including IL-8, in AhR high expressing tumors (Vacher et al., 2018). We demonstrated that C/EBPβ serves as a key transactivator for AhR-mediated COX-2 gene induction (Vogel et al., 2000; Vogel et al., 2004). Interestingly, COX-2, CXCL1, and IL-8 have been identified as critical genes that mediate breast cancer invasion and metastasis to lung and lymph nodes (Freund et al., 2003; Minn et al., 2005; Ahmed et al., 2021). A recent report suggests that inhibition of COX-2 expression reduces mammary tumor multiplicity and size in the polyoma middle T antigen (PyMT) mouse model (Esbona et al., 2016). In our recent study we demonstrated that overexpression of AhRR (Aryl Hydrocarbon Receptor Repressor) suppresses AhR-driven (TCDD-stimulated) growth of syngeneic mammary tumors as well the onset, growth and metastasis of spontaneous mammary tumors in PyMT mice (Vogel et al., 2021). In human breast cancer, high expression of AhRR, the dedicated AhR repressor, independently predicts prolonged metastasis-free survival (Vacher et al., 2018), in agreement with our findings in PyMT mice (Vogel et al., 2021). Interestingly, knockdown of AhRR in normal human mammary epithelial cells resulted in anchorage-independent cell growth suggesting that the AhRR may function as a tumor suppressor gene (Zudaire et al., 2008).

In a mouse model of BRCA1-associated breast cancer, AhR was found to transcriptionally induce the EGF receptor ligand, Amphiregulin, driving tumor growth and macrophage infiltration. Of note, this was inhibited by the combination of an AhR inhibitor and an EGF receptor inhibitor, suggesting new therapeutic possibilities for this type of breast cancer (Kubli et al., 2019). The relationship between AhR activation and breast cancer-related death was recently assessed using an artificial intelligence tool to analyze the scientific literature, with strong evidence that AhR activation is an adverse outcome pathway in breast cancer (Benoit et al., 2022).

Interestingly, many studies have also provided evidence for a tumor suppressor role for AhR, with evidence that AhR can inhibit tumor growth (Fritz et al., 2007; Jin et al., 2014; Feng et al., 2020) while inhibition of AhR or AhR deficiency promotes tumor development (Abdelrahim et al., 2003; Safe et al., 2017). For example, in the ApcS580/+; KrasG12D/+ mouse model of colon tumorigenesis, intestinal epithelial specific AhR knockout promoted tumorigenesis through enhanced Wnt signaling (Han et al., 2021). In p53 deficient mice, AhR knockout significantly increased incidence of thymic lymphomas and sarcomas and decreased survival (Phillips et al., 2022). In a mouse model of sonic hedgehog type-medulloblastoma, AhR deletion in cerebellar granule cell progenitors accelerated tumorigenesis through increased TGFβ-SMAD3 signaling (Sarić et al., 2020) with high AhRR expression linked to decreased patient survival. Further, in an unbiased functional genomics screen, AhR was identified as metastasis suppressor in a lung cancer model (Nothdurft et al., 2020). In in vitro studies, AhR was demonstrated to cooperate with the Rb tumor suppressor to prevent S-phase cell cycle entry (Puga et al., 2000) while activation of AhR by the prototypical ligand TCDD inhibited the growth of MCF7 breast cancer cells (Vogel and Abel 1995). David Sherr’s team investigated AhR agonists and antagonists in a direct comparison and concluded that the sustained activation of AhR drives the later, more lethal stages of some cancers, but that AhR agonists under some circumstances can counteract tumor development and may also serve as cancer therapeutics (Narasimhan et al., 2018). In this vein, O’Donnell (O’Donnell et al., 2021) and others (Rowland et al., 2019) have pursued SMAhRTs, Select Modulators of AhR-regulated Transcription, to specifically exploit the anti-cancer functions of AhR. Notably, they identified a modulator which induced AhR-dependent Fas ligand expression and breast and liver tumor cell apoptosis without increasing expression of the prototypical AhR target gene, CYP1A1, suggesting that AhR transcriptional activity can be fine-tuned, to specifically unlock its function as a tumor suppressor.

## Cytokines and chemokines in breast cancer and the tumor microenvironment

**FIGURE 1:**
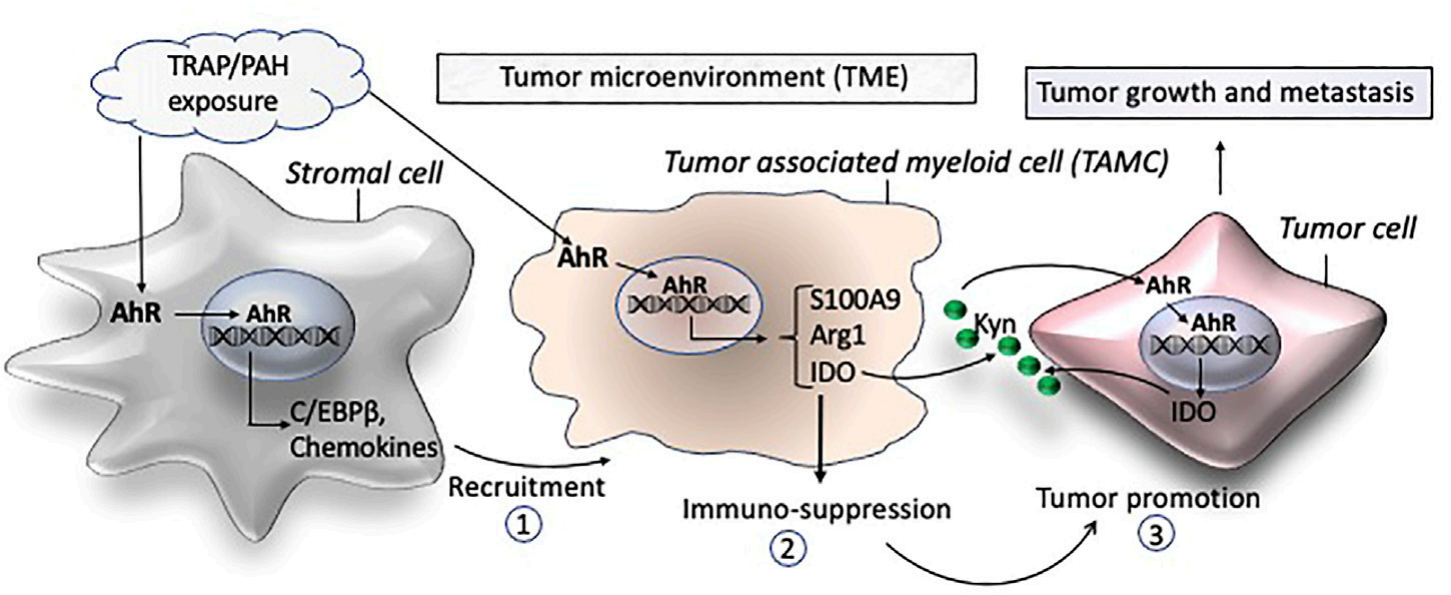
Schematic of proposed mechanisms by which traffic-associated air pollution (TRAP) activates AhR to induce the accumulation of TAMCs (1) and expression of immune suppressive factors (2) leading to a tumor promoting microenvironment (3) and growth and metastasis of breast cancer.

The tumor microenvironment (TME) corresponds to the fact that tumor cells are surrounded in close proximity by a number of non - cancerous cells including cancer associated fibroblasts (CAFs), mesenchymal stem cells (MSCs), adipocytes, myeloid-derived suppressor cells (MDSCs), tumor associated macrophages (TAMs), tumor associated neutrophils (TANs), tumor infiltrating lymphocytes (TILs), and endothelial cells (Joyce and Pollard 2009; Lazennec and Lam 2016; Binnewies et al., 2018). In addition to direct contact with tumor cells, TME cells will interact with tumor cells though a number of different soluble factors including cytokines and chemokines, which will reshape TME to support cancer initiation, progression, and metastasis (Ali and Lazennec 2007; Lazennec and Richmond 2010; Mancini et al., 2021) (Figure 1).

In breast cancer, many chemokines and cytokines have been analyzed and identified as important factors contributing to the development of breast tumors (Narita et al., 2016; Masih et al., 2022). In particular the CXCR4/CXCL12 axes has been reported to control breast cancer metastasis and the involvement of CAFs (Muller et al., 2001; Orimo et al., 2005). The CAF-driven CXCR4/CXCL12 axis may also stimulate the accumulation of protumorigenic lipid associated macrophages which supports an immunosuppressive microenvironment in breast cancer (Timperi et al., 2022).

CCL2 and CCL5 have also retained attention in breast cancer, as they are expressed by cancer cells and promote the recruitment of TAMs and metastasis by inducing Th2 polarization of CD4^+^ T cells (Chavey et al., 2007; Soria and Ben-Baruch 2008; Zhang et al., 2015; Brummer et al., 2018). In addition, the ligands of CXCR2 (CXCL1, 2, 3, 5, 6, 7, 8) have been shown in a number of studies to be involved in the aggressiveness of triple negative breast cancers (TNBC) (Bieche et al., 2007; Chavey et al., 2007; Acharyya et al., 2012). The genes of these chemokines are encoded by a small region of chromosome 4q21 and have been found to be coregulated in TNBC (Bieche et al., 2007). Moreover, cancer cells expressing high levels of CXCL1 and CXCL2 acquire an advantage in terms of survival in metastatic sites and favor the recruitment of TANs (Acharyya et al., 2012). Interestingly, CXCR2 itself is also playing a major role in the aggressiveness of TNBC, in particular through its expression on TANs (Boissiere-Michot et al., 2020; Boissiere-Michot et al., 2021). Although the levels of CXCR2-expressing neutrophils is correlated to high grade breast cancers, its role is rather to counteract tumor progression (Boissiere-Michot et al., 2021), as it is correlated with a better survival of the patients and its deletion favors tumor growth and metastasis (Timaxian et al., 2021). There are many links between AhR and CXCR ligands in particular. For instance, we have shown that the complex of AhR and NFkB RelB was able to bind to a specific binding elements of chemokines including the CXCL8 promoter and to promote its activation though protein kinase A (Vogel et al., 2007). RelB/AhR complex is also involved in the overexpression of CXCL8 in breast cancer (Vogel et al., 2011; Bekki et al., 2015). A significant elevated level of CXCL8 mRNAs expression (56-fold) has also been found in tissue samples of high stage compared to low stage patients and adipose-derived stem cells (Razmkhah et al., 2010). AhR may also interact with NFkB RelA causing the upregulation of c-myc and stimulation of tumorigenesis in MCF-7 cells (Kim et al., 2000). Further HER2 overexpression in MCF-7 cells resulted in pro-inflammatory signaling and induction of IL-6 enhancing mammosphere formation in an AhR-dependent manner (Zhao et al., 2013). The role of AhR as a mediator of chronic inflammation in breast cancer has been recently reviewed elsewhere (Guarnieri 2020). Moreover, a recent study by Kubli et al. has shown that AhR was induced by reactive oxygen species (ROS) in mammary epithelial cells, which in turn enhance AREG (amphiregulin) production (Kubli et al., 2019). In basal-like and BRCA1-related breast cancers, ROS expression was correlated with AhR levels and the expression of the chemokines CXCL1, CXCL2, and CCL5. Targeting AhR or AREG reduced the recruitment of macrophages in tumors in mouse models and AREG expression was associated with the density of macrophages in human tumors. Another cytokine upregulated by AhR activation is IL-22 which is an important factor controlling host defense and gut immunity. However, dysregulation of IL-22 may contribute to the development of TNBC and the pathology in breast cancer (Kim et al., 2014; Voigt et al., 2017; Wang et al., 2018; Katara et al., 2020). IL-22 has also been described to mediate macrophage infiltration in the TME and the migration of breast cancer cells (Kim et al., 2020). Results from MCF-7 cells co-cultured with preadipocytes and an *in vivo* zebrafish model showed that prototypical AhR ligand TCDD enhanced the invasive and metastatic potential of MCF-7 cells implicating the importance of AhR in the TME (Koual et al., 2021).

## AhR as a critical player in the tumor microenvironment of breast cancer

The development of metastatic disease, which accounts for greater than 90% of cancer mortality, requires collaboration between tumor cells and their environment. Recent studies reveal that the TME possesses remarkable cellular heterogeneity with an important role of immune cells in the development and progression of breast cancer (Ben-Baruch 2003; Place et al., 2011). The TME also consists of an acellular component (e.g., soluble cytokines, chemokines, and growth factors), that forms part of the stromal structure as described above. TAMs and MDSCs are tumor-associated myeloid cells (TAMCs) and have been identified as key players in breast cancer progression and metastasis (Cha and Koo 2020). MDSCs are myeloid cells at earlier stages of differentiation and serve as precursor of TAMs (Coffelt et al., 2009). Their presence and frequency have been directly correlated with tumor aggressiveness and is associated with poor survival rates in breast cancer (Leek et al., 1996; Mukhtar et al., 2011a; Mahmoud et al., 2012; Zhang et al., 2012; Zhao et al., 2017; Qiu et al., 2018). They have been found to drive cancer progression *via* immune regulatory functions creating a tolerogenic environment allowing the tumor to progress (Figure 1). TAMCs inhibit tumor immune responses by blocking T cell functions and proliferation, but they also directly trigger tumor growth by promoting cancer stemness, angiogenesis, EMT and metastasis formation. In breast cancer patients, levels of MDSCs in peripheral blood were found to be about 10-fold higher compared to healthy control individuals (Safarzadeh et al., 2019). Moreover, they found a direct relationship between MDSC levels and tumor stage of breast cancer patients. The study underlines the importance of MDSCs in tumor progression and invasion which was supported by Diaz-Montero et al. (Diaz-Montero et al., 2009) showing that MDSC levels are associated with the clinical stage and metastatic disease burden in patients with breast cancer. MDSCs possess strong immunosuppressive activities and interact with other immune cells to regulate their functions. The number and abundance of TAMs and MDSCs is considered to be an important factor in the clinical success of cancer immunotherapy, underlining their critical role in suppression of immunity in breast cancer patients (Gnant et al., 2011; Gomez-Roca et al., 2015).

AhR plays a critical role in carcinogenesis and tumor immunity (Murray et al., 2014; Xue et al., 2018). Activation of AhR *via* Kynurenine (Kyn) produced by the immunosuppressive enzyme indoleamine 2, 3-dioxygenase (IDO) in glioblastoma cells has been found to induce the accumulation of TAMs (Takenaka et al., 2019; McKay et al., 2021). They reported that the AhR ligand Kyn is able to activate AhR in TAMs, leading to an increased expression of the chemokine receptor CCR2 by TAMs, which enhances the recruitment of TAMs in response to CCL2. Moreover, AhR stimulates the production of the exonucleotidase CD39 by TAMs, interfering with the function of cytotoxic CD8^+^ T cells (Takenaka et al., 2019). In melanoma patients, high levels of IDO1 are associated with high levels of Kyn and immunosuppression (Campesato et al., 2020). Using a melanoma model, it was shown that tumors expressing high levels of IDO1 present an enrichment of TAMs and selective inhibition of AhR decreases tumor progression, by inhibiting the immunosuppression mediated by IDO1. Another link of AhR with immune response in cancer is highlighted by the fact that AhR mediates the induction of the poliovirus receptor CD155 by IL-4 and LPS in macrophages, as CD155 is suppressing T cell function (McKay et al., 2021). In the same line, the inhibition of AhR activity in a model of pancreatic cancer promotes the infiltration of CD8^+^ T cells and improves the response to immune therapy (Hezaveh et al., 2022). This study also showed that AhR is highly expressed in TAMs, involved in their polarization, and associated with a reduction of iNOS, CCL4, and TNFα levels. Further, Neamah et al. (2019) found that treatment with the AhR ligand TCDD induces the accumulation of MDSCs in the peritoneal cavity. Interestingly, we found an accumulation of CD11b+ F4/80+ and CD11b+ F4/80- Ly6G + cell subsets in adipose tissue associated with a significant increase of the chemokine CXCL5 in TCDD-treated mice (Vogel et al., 2016) which indicates accumulation of TAMs and MDSCs (Ugel et al., 2015). Although TAMs and MDSCs are regarded as separate populations, some markers including CD11b are shared among TAMs and MDSCs (Ugel et al., 2015). There are specific markers (e.g., Ly6G and Ly6C) that can be used to distinguish them. Further, MDSCs and TANs express high levels of S100A9 and the immunosuppressive enzymes IDO and arginase 1 (Arg1) which are specific for their immune-suppressive activity in TME of breast cancer (Fridlender et al., 2009; Ostrand-Rosenberg 2016).

The polarization of TAMs and MDSCs within the TME is highly dependent on the local milieu of immune regulatory factors (e.g., C/EBPβ and S100A9) and cytokines and chemokines which can originate from stromal cells (Figure 1). Recently, we identified C/EBPβ as a critical transcription factor in AhR-dependent induction of S100A9 after treatment with PM rich in PAHs (Dahlem et al., 2020). The S100 calcium binding protein S100A9 has been shown to play a critical role in mediating the expansion of MDSCs in breast cancer models (Zhao F et al., 2012). Moreover, S100A9 can act as a transcriptional coactivator during breast cancer development (Song and Struhl 2021) and promotes the immune-suppressive activity of MDSCs (Ostrand-Rosenberg 2016). Regardless of any direct lineage link and distinction between MDSCs, TANs and TAMs, the most important criteria for their role in carcinogenesis are their immune-suppressive and pro-tumoral activities. Importantly, the AhR has been demonstrated to regulate the expression of immune-regulatory markers including Arg1, IDO, IL-10, COX-2, C/EBPβ, and S100A9 (Vogel et al., 2008; Bankoti et al., 2010; Benson and Shepherd 2011; Simones and Shepherd 2011; Vogel et al., 2013; Neamah et al., 2019; Dahlem et al., 2020), which are critical factors in the pathogenesis of breast cancer (Yu et al., 2013; Yu et al., 2014; Dey et al., 2021). Moreover, TCDD increased the activity of the immunosuppressive enzyme IDO which mediates tumor immunity in breast cancer cells (Bekki et al., 2015). Interestingly, AhR as well as NFkB RelB have been shown to induce IDO expression (Vogel et al., 2008; Yu et al., 2014), which is also critically involved in the immunosuppressive mechanisms of myeloid-derived suppressor cells (MDSCs) in breast cancer (Yu et al., 2013). The number and frequency of TAMs and MDSCs have been directly correlated with tumor aggressiveness, and indirectly correlated with clinical outcome in breast cancer (Mukhtar et al., 2011b). The literature also shows that accumulation of TAMCs is a significant prognostic factor in breast cancer (Zhao et al., 2017). A significant heterogeneity of TAMCs in mammary tumors has been described (Movahedi et al., 2010) and the activation of AhR has been shown to activate TAMs (Takenaka et al., 2019) and induce the accumulation of MDSCs (Neamah et al., 2019). The mechanisms that are driving the polarization of immune-suppressive TAMCs in the TME by AhR signaling activated through the exposure to PM, PAHs, and dioxin like chemicals are not clear yet. In summary, data from the literature strongly suggest AhR’s critical role in the microenvironment of mammary tumorigenesis promoting tumor progression and metastasis.

## The intersection between environmental exposure and cancer stemness

Breast cancer stem cells (BCSCs), a small but highly plastic subpopulation of tumor cells, have taken center stage in the interplay between chemoresistance, recurrence, and metastasis (Shan et al., 2021). BCSCs, capable of both self-renewal and recapitulation of tumor heterogeneity, are multidrug-resistant (MDR) and highly immune-evasive. MDR is due in part to robust expression of the ABCG2 efflux protein, also known as Breast Cancer Resistance Protein (BCRP) (Zhou et al., 2001; Zattoni et al., 2022), a direct transcriptional target of AhR (Tan et al., 2010). Substantial efforts have focused on strategies which will lead to the effective elimination of BCSCs, however it is recognized that standard endocrine and chemotherapy regimens paradoxically enrich for BCSCs with mesenchymal features, driving tumor recurrence (Li et al., 2008; Creighton et al., 2009; Famta et al., 2022).

AhR has been implicated in cancer stemness and immune evasion in various tumor types serving as a “molecular bridge” between environmental exposure and poor patient prognosis. In lung and nasopharyngeal carcinoma cells, AhR was shown to drive the expression of a panel of stemness genes, including ABCG2 (Yan et al., 2018). Interestingly, ABCG2 has been directly implicated in expanding the stem population in osteosarcoma cells (Zhou et al., 2001). In non-small cell lung carcinoma, the deubiquitinase UCHL3 promoted cancer stemness through stabilization of AhR (Ouyang et al., 2020). Recently, activation of AhR by the endogenous ligand kynurenine was linked to colon cancer stemness, immune evasion through PD-L1 induction and metastasis (Miyazaki et al., 2022). In an oral squamous cell carcinoma model, tumor cell- and immune cell-expressed AhR collaborated to promote tumor immune evasion with AhR knockout in tumor cells restoring anti-tumor immunity (Kenison et al., 2021).

In breast cancer, tranilast, a tryptophan metabolite and AhR agonist, was shown to inhibit the BCSC population in MDA-MB-231 (triple negative) breast cancer cells and abrogate metastasis in a tail vein injection model (Prud’homme et al., 2010), in an AhR dependent manner. In agreement with these findings, several studies reported that AhR activation inhibits the BCSC population (Saito et al., 2021; Yamashita et al., 2021). In MCF7 (ER+) cells expressing a constitutively active AhR or treated with the AhR agonists 3-Methylcholanthrene (3 MC) or β-naphthoflavone (β-NF), the BCSC population was decreased (Zhao S et al., 2012). Most recently, camalexin, an indole phytoalexin and AhR agonist was shown to decrease the BCSC population of MCF7 and T47D (ER+) breast cancer cells (Yamashita et al., 2022). Conversely, AhR activation by the potent agonists TCDD and DMBA was found to increase the breast cancer stem cell population and was implicated in doxorubicin resistance of MCF-7 breast cancer cells (Al-Dhfyan et al., 2017). In Tamoxifen-resistant MCF7 cells, AhR antagonism inhibited the BCSC population and also inhibited tumor growth (Dubrovska et al., 2012). In Hs578T (triple negative) and SUM149 (inflammatory) breast cancer cells, AhR was shown to augment the BCSC population, and its inhibition decreased tumor growth and sensitized cells to both adriamycin and paclitaxel (Stanford et al., 2016). This study also found a significant correlation between AhR activity and “cancer stem cell- and migration/invasion-associated gene sets” in an analysis of 79 human breast cancer cells lines and more than 1,850 human breast cancers. In inflammatory breast cancer, AhR was linked to BCSC maintenance through the Wnt5a/β-catenin signaling pathway (Mohamed et al., 2018). AhR crosstalk with Wnt/β-catenin signaling in the regulation of CSCs has been reported in several studies (Al-Dhfyan et al., 2017; Akhtar et al., 2022).

The role of AhR in cancer stemness and breast cancer stemness more specifically is complex, influenced by mode of AhR activation, engagement with various signaling pathways and cell context. Nevertheless, the collective evidence strongly suggests that AhR activation by environmental toxins and endogenous ligands (Ala 2021) aligns with chemoresistance, recurrence and metastasis, the hallmarks of cancer stemness. This places AhR at the intersection between racial/ethnic and socioeconomic disparities in toxin exposure in under-resourced neighborhoods, as discussed previously, and cancer stemness, undermining response to cancer therapy, worsening the riskscape that an individual must navigate. In a recent review by Lagunas-Rangel, the authors pose the question “Can Exposure to Environmental Pollutants Be Associated with Less Effective Chemotherapy in Cancer Patients?” The authors summarize evidence which strongly supports this hypothesis, which includes toxins which activate AhR (Lagunas-Rangel et al., 2022). Therachiyil examines this from the perspective of gynecological cancers. (Therachiyil et al., 2022).

## Conclusion

**FIGURE 2:**
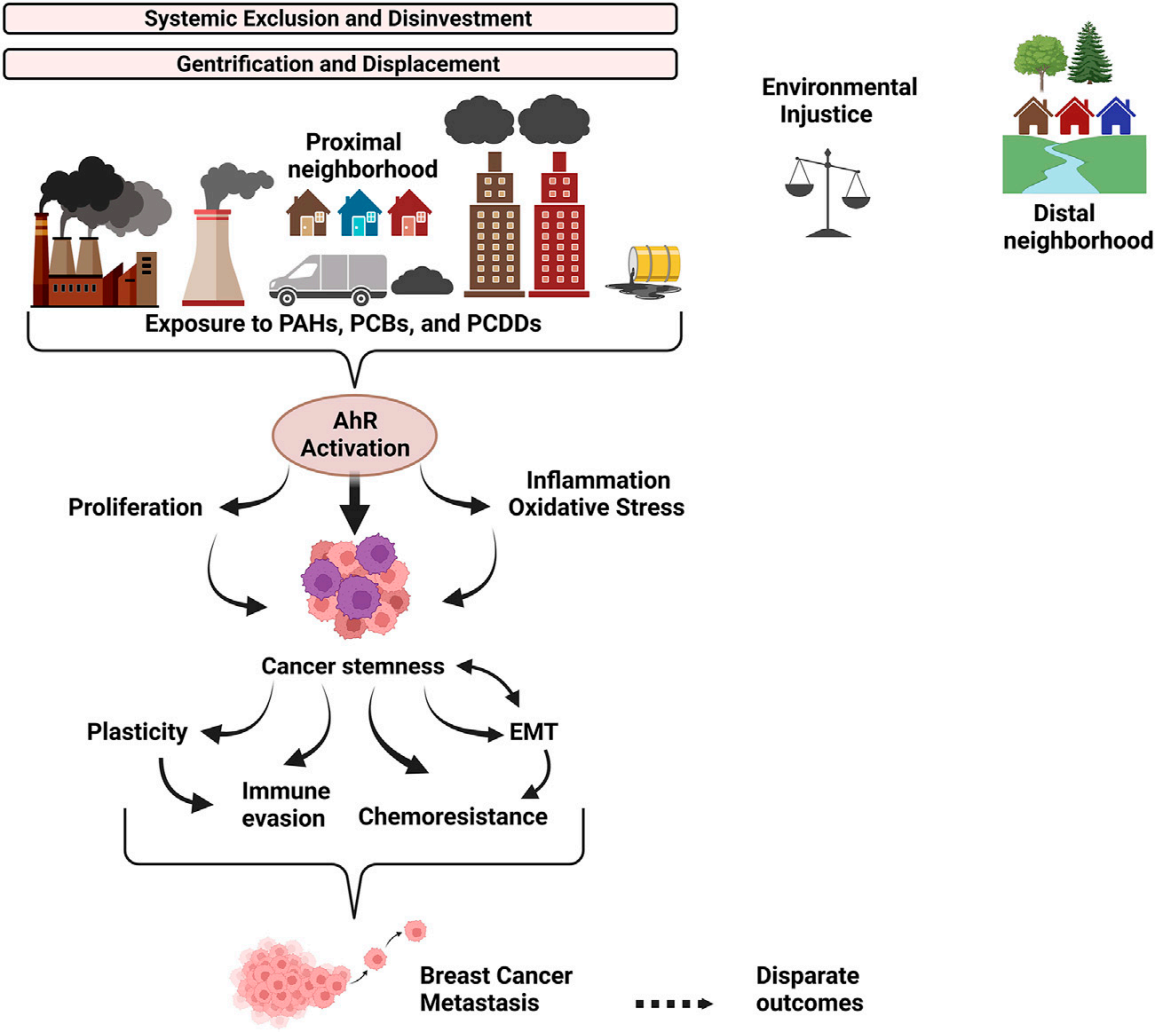
Overview of the link between systemic exclusion, environmental injustice, and AhR-driven tumor biology. Discriminatory housing and lending policies (ex: redlining) drove neighborhood racial/ethnic and socioeconomic segregation which persists today due to ongoing systemic discrimination and gentrification. Resources were and are disproportionately allocated to wealthier neighborhoods, contributing to neighborhood disinvestment. The proximal neighborhood has more traffic, pollution-generating factories and dump sites. It has less green space for stress relief and exercise, worsening the riskscape that individuals in the proximal neighborhood must navigate. Individuals living in the proximal neighborhood are chronically exposed to environmental toxins, tipping the scales of environmental justice against them. Polycyclic aromatic hydrocarbons (PAHs), Polychlorinated dibenzo-p-dioxins (PCDDs), and Polychlorinated biphenyls (PCBs) are generated by combustion processes as components of ambient particulate matter (PM) derived from urban areas and industrial activities. PAHs, PCDDs and PCBs robustly activate the AhR signaling pathway, promoting cancer stemness and interrelated functional outcomes, including plasticity, chemoresistance, EMT and immune evasion, which synergize to drive breast cancer metastasis and disparate outcomes for individuals in proximal neighborhoods.

Collectively, the body of literature indicates that the role of AhR in cancer is complex, with ample evidence for both an oncogenic and tumor suppressor function, depending on cell and tissue context and mode of AhR activation. However, exposure studies indicate that environmental pollutant-mediated activation of AhR is consistently oncogenic, highlighting the potential for cautious therapeutic intervention. The data from human and *in vivo* studies, as well as *in vitro* experiments suggest that exposure to environmental pollutants especially PAHs and dioxin-like chemicals, potent ligands for AhR, increases breast cancer risk and worsens outcome through chemoresistance, immune evasion, EMT, tumor cell proliferation, and metastasis, linked functional outcomes of cancer stemness (Figure 2). Some critical questions remain, including how AhR activation modulates the tumor microenvironment. This review also highlights the role of AhR at the interface between historical and existing systemic practices - which reinforce residential segregation and environmental injustice - and the molecular drivers of aggressive tumor biology. While policies and molecules are not frequently in the same conversation, greater dialogue is needed and opportunities for “upstream” disease prevention through systemic change should be prioritized.
